# ID 314 - Benefícios na Incorporação de Duas Novas Tecnologias ao Programa Saúde da Mulher no SUS

**DOI:** 10.5327/2237-9622.2025.v34s1.314

**Published:** 2025-11-25

**Authors:** Rafael Poloni, Poliana Bernardes Gonçalves, Herbênio Elias Pereira, Daniela Dias Dantas, Melina Calmon Silva, Marco Aurélio Pereira

**Keywords:** Programa Saúde da Mulher, Assistência Farmacêutica, Atenção Primária à Saúde, política de saúde, orçamento

## Abstract

**Introdução:**

O Ministério da Saúde coordena a Política Nacional de Atenção Integral à Saúde das Mulheres (Pnaism), que promove a melhoria das condições de vida e saúde das mulheres brasileiras, com garantia de direitos e ampliação da promoção, prevenção, assistência e recuperação da saúde; contribui para a redução da morbidade e mortalidade feminina no Brasil; e amplia, qualifica e humaniza a atenção integral à saúde da mulher no Sistema Único de Saúde (SUS).

Uma das estratégias para garantir o acesso das mulheres às tecnologias em saúde é por meio do Componente Básico da Assistência Farmacêutica (Cbaf), que disponibiliza medicamentos anticoncepcionais e insumos do Programa Saúde da Mulher na Atenção Primária à Saúde. A aquisição desses itens à população, no âmbito do Cbaf, é de responsabilidade do Ministério da Saúde.

Em 2021, houve solicitação do Ministério da Saúde à Comissão Nacional de Incorporação de Tecnologias no Sistema Único de Saúde (Conitec) de avaliação da incorporação de novos contraceptivos – os injetáveis mensais acetato de medroxiprogesterona + cipionato de estradiol (25 mg + 5 mg) e/ou algestona acetofenida + estradiol enantato (150 mg + 10 mg) para mulheres em idade fértil – com o intuito de ampliar o acesso da população às tecnologias eficazes, seguras e de qualidade, como alternativas ao medicamento já disponível desde 2004 no SUS, o enantato de noretisterona + valerato de estradiol (50 + 5) mg/mL.

Isto posto, este trabalho objetiva apresentar os benefícios trazidos pela incorporação dessas novas tecnologias ao SUS.

**Método:**

Os dados foram coletados em fontes oficiais e de domínio público do Ministério da Saúde.

**Resultados:**

Em 2023, foram adquiridas 6.680.900 unidades do noretisterona + estradiol, por meio do Contrato n.º 89, de 3 de maio de 2023, com valor unitário de R$ 13,39 (total: R$ 89.577.761,00), e medroxiprogesterona + estradiol por meio do Contrato n.º 94, de 25 de maio de 2023, no quantitativo de 329.424 e valor unitário de R$ 12,60 (total: R$ 4.150.742,00). Em 2024, foram adquiridas 6.317.407 unidades de noretisterona + estradiol (Contratos n.º 120, de 5 de abril de 2024, e n.º 127, de 8 de abril de 2024), com valor unitário de R$ 4,68 (total: R$ 29.565.675,36). O algestona acetofenida, em 2024, por meio do Contrato n.º 109, de 5 de março de 2024, foi adquirido em quantitativo de 368.134 com valor unitário de R$ 8,11 (total: R$ 2.985.566,74). Houve queda drástica (65%) do valor unitário do enantato de noretisterona, que impactou diretamente o valor dos contratos em 2024. Em termos de impacto orçamentário, a redução do custo unitário em 2024 desse item trouxe uma significativa otimização de recursos pelo Ministério da Saúde, de pelo menos R$ 60 milhões, o que gera eficiência da Administração Pública.

**Conclusão:**

A Conitec apontou, no Relatório de Recomendação n.º 724/2022, os benefícios da incorporação de duas novas tecnologias para a contracepção, incluindo a ampliação do acesso de opções terapêuticas. Em decorrência dessa incorporação ao SUS dos novos anticoncepcionais injetáveis mensais, houve aumento da concorrência nas aquisições públicas, com redução significativa do preço da tecnologia já disponibilizada no SUS, gerando economia ao erário, o que possibilitou a aquisição das novas tecnologias incorporadas. Essa estratégia trouxe benefícios orçamentários, com otimização e eficiência do gasto público, estímulo ao mercado desses itens, bem como ampliação de acesso à população a tecnologias seguras, eficazes e de qualidade.

